# Adults with Crohn’s disease exhibit elevated gynoid fat and reduced android fat irrespective of disease relapse or remission

**DOI:** 10.1038/s41598-021-98798-9

**Published:** 2021-09-28

**Authors:** Lisa Dowling, Philip Jakeman, Catherine Norton, Maeve M. Skelly, Hamid Yousuf, Miranda G. Kiernan, Margaret Toomey, Sheila Bowers, Suzanne S. Dunne, J. Calvin Coffey, Colum P. Dunne

**Affiliations:** 1 Centre for Interventions in Infection, Inflammation and Immunity (4i), Limerick, Ireland; 2 School of Medicine, University of Limerick, Limerick, Ireland; 3 Physical Education and Sports Sciences, University of Limerick, Limerick, Ireland; 4 Department of Gastroenterology, University Limerick Hospital Group, Limerick, Ireland; 5 Department of Dietetics, University Limerick Hospital Group, Limerick, Ireland; 6 Department of Surgery, University Limerick Hospital Group, Limerick, Ireland

**Keywords:** Gastroenterology, Gastrointestinal diseases, Inflammatory bowel disease, Crohn's disease

## Abstract

Crohn’s disease (CD) is a debilitating inflammatory bowel condition of unknown aetiology that is growing in prevalence globally. Large-scale studies have determined associations between female obesity or low body mass index (BMI) with risk of CD at all ages or 8– < 40 years, respectively. For males, low BMI entering adult life is associated with increased incidence of CD or ulcerative colitis up to 40 years later. Body composition analysis has shown that combinations of lean tissue loss and high visceral fat predict poor CD outcomes. Here, we assessed dietary intake, physical activity and whole or regional body composition of patients with CD relapse or remission. This anthropometric approach found people with CD, irrespective of relapse or remission, differed from a large representative healthy population sample in exhibiting elevated gynoid fat and reduced android fat. CD is associated with mesenteric adipose tissue, or “creeping fat”, that envelops affected intestine exclusive of other tissue; that fat is localised to the android region of the body. In this context, CD mesenteric adiposity represents a stark juxtaposition of organ-specific and regional adiposity. Although our study population was relatively small, we suggest tentatively that there is a rationale to refer to Crohn’s disease as a fatty intestine condition, akin to fatty liver conditions. We suggest that our data provide early insight into a subject that potentially warrants further investigation across a larger patient cohort.

## Introduction

Retrospective analysis of body mass index (BMI) records, notably even at population levels, has determined associations between female obesity or low BMI with risk of Crohn’s disease (CD) at all ages or 8– < 40 years, respectively^1^. For males, low BMI entering adult life is associated with an increased incidence of CD or ulcerative colitis up to 40 years later^2,3^. In adulthood, patients with CD often present as overweight or obese, a trend that is consistent with that of the general population as, for example, Nic Suibhne et al. reported 40% of patients with CD (n = 100) were overweight/obese, compared to 52% in a control group (n = 100)^4^, while Sousa-Guerreiro et al*.* reported a BMI of > 25 kg/m^2^ in 32% and 33.8% of a CD cohort and healthy control group, respectively^5^.

However, BMI is considered only a rough guide to body composition when applied at the population level, and has the potential to misclassify at the individual level (Toomey et al. 2015)^6^. Far more accurate measurement of whole body and regional body composition can be provided by the 3-component (fat, lean and bone) model of body composition generated by dual energy x-ray absorptiometry (DEXA). Such 3-component modelling of body composition has provided normative population data^7^, for athletes^8^ and for patient specific reference data: e.g., osteoporosis^9^ and diabetes^10^.

While initial DEXA studies of Crohn’s disease were performed in the 1990s^11^, multiple reports substantiate that CD is associated with lower lean mass and higher fat mass in the CD setting independent of BMI^12^. More specifically, low muscle attenuation and higher visceral fat index appear to be associated with more severe CD phenotypes^13^, and Bryant et al. found that increasing obesity in CD coincided with a decrease in lean muscle mass over time^14^. Boparai et al. investigated this inverse relationship further, utilising skeletal muscle indices and differential analysis of visceral and subcutaneous fat to determine that combination of sarcopenia and high visceral fat predicts poor outcomes in patients with CD^15^. In addition, as lean muscle mass has been shown to be of more importance than fat mass in determining bone density, the observed loss of lean muscle mass in CD cohorts could have a significant impact on bone health^16^.

Therefore, it is unsurprising that emphasis has been placed increasingly on understanding the role of elevated fat levels in the physiology of CD and, potentially, in its aetiology and treatment. In particular, there has been a focus on mesenteric adipose tissue in CD^17^. Also referred to as “creeping fat”, this layer of tissue has been thought of as a physical barrier to inflammation & inflammatory markers, controlling host immune response to translocation of gut bacteria, and immunomodulation^18,19^. It is now, however, evident that mesenteric fat is implicated in both pathogenesis and treatment outcomes in CD^17,20^ especially disease activity and severity of symptoms^21^, bowel damage and stricture formation^20,22,23^.

In that context, acknowledging that changes in disease presentation, clinical management, nutrition and/or physical activity of patients with CD manifest as changes in body composition^12^, our objectives were to investigate the tendency towards lower lean mass and higher fat mass suggested previously. In a cohort of patients in either relapse or remission we further aimed to evaluate the influence of dietary intake and physical activity on their body composition, determined through whole body analysis and an innovative approach to regional body composition.

## Results

### Crohn’s disease cohort

Of the participating CD patient cohort, 16 had remitting disease and 14 were undergoing relapse. The median CDAI was 143 (61) for all participants, 130 (45) for males and 143 (61) for females. Three participants had an ileostomy (Table 1).

**Table 1:** Clinical details of participants including gender, age, disease activity, disease location and disease behaviour.

Characteristics	All		Male		Female	
	(n = 30)		(n = 13)		(n = 17)	
Age, years, mean (SD)	43.1	(13.5)	44.7	(15.2)	41.9	(12.4)
Disease duration, years, median (IQR)^a^	7.0*	(3.0)	9.0*	(5.0)	6.0*	(3.3)
CDAI, score, median (IQR)^b^	143*	(61)	130*	(45)	143	(61)
Remission ≤ 150 (n)	16		7		9	
Active disease > 150 (n)	14		6		8	
Harvey Bradshaw Index, score, mean (SD)	5	(3)	5	(2)	6	(4)
Remission < 5 (n)	13		6		7	
Mild disease 5–7 (n)	8		6		2	
Moderate disease 8–16 (n)	9		1		8	
Severe disease > 16 (n)	0		0		0	
Age at onset (n)						
A1: less than 16 years	2		2		0	
A2: between 17 and 40 years	21		8		13	
A3: over 40 years	7		3		4	
Disease location (n)						
L1: ileal	12		2		10	
L2: colonic	3		1		2	
L3: ileocolonic	15		10		5	
Disease behaviour (n)						
B1: non-stricturing, non-penetrating	15		7		8	
B2: stricturing	4		1		3	
B3: penetrating	7		2		5	
P: perianal disease	4		3		1	
Stoma in situ (n)	3		2		1	
Previous surgery for CD (n)	13		4		9	
Medications**:						
Aminosalicylates (5-ASA)	9		3		6	
Corticosteroids	10		7		3	
Immunomodulators						
Azothioprine	11		6		5	
6-mercaptopurine	7		4		3	
Antibiotics	3		2		1	
Biologics						
Infliximab	5		2		3	
Adalimumab	8		4		4	

CDAI, Crohn’s disease Activity Index. ^a^n = 29 with information on years of diagnosis; ^b^n = 29 with CDAI; *not normally distributed (Shapiro–Wilk *p* < 0.05); **There was no observed association between medical treatment and either physical activity levels or fat deposition.

### Body composition

Body composition analysis determined no significant differences between remitting and relapsing participants. Therefore, the full patient cohort was compared with the general healthy Irish population reflected in the ULBC. Notably, the male patients had significantly higher BMI (28.1 (4.9) kg/m^2^) compared with the ULBC (25.1 (3.9) kg/m^2^) (*p* = 0.016), while female BMI (27.0 (10.0) kg/m^2^) was similar to ULBC (24.2 (4.9) kg/m^2^). BFM of male participants (29.7 (8.6) kg) were significantly greater than counterparts in the ULBC (16.6 (10.8) kg) (*p* < 0.005). Similarly, when adjusted for stature, BFMI was higher in males with CD (9.6 (2.9) kg/m^2^) than those of the ULBC (5.2 (3.5) kg/m^2^; *p* < 0.005). Indices of ALTM and LTM were similar between male and female CD participants and ULBC (Table 2).

**Table 2:** Body compositional measurements of participants compared with ULBC.

		ULBC Study		Patients with CD		*P value*
**BM**	Males	80.8†	(14.7)	88.9	(16.5)	0.101
(kg)	Females	65.1†	(13.5)	69.0†	(30.5)	0.356
**Height**	Males	1.8	(0.1)	1.8	(0.1)	0.626
(m)	Females	1.6†	(0.1)	1.6	(0.1)	0.887
**BMI**	Males	25.1†	(3.9)	28.1*	(4.9)	0.016
(kg/m^2^)	Females	24.2†	(4.9)	27.0†	(10)	0.227
**LTM**	Males	61.3	(7.1)	56.0	(9.3)	0.063
(kg)	Females	40.5†	(6.3)	39.8	(6.7)	0.554
**LTMI**	Males	18.9†	(2.2)	17.7	(2.6)	0.248
(kg/m^2^)	Females	15.1†	(1.7)	15.0	(2.1)	0.652
**ALTM**	Males	30.0	(4.0)	27.2	(5.4)	0.088
(kg)	Females	18.2†	(3.3)	18.1	(3.7)	0.722
**ALTMI**	Males	9.3	(1.0)	8.7	(1.6)	0.181
(kg/m^2^)	Females	6.8†	(1.0)	6.7	(1.2)	0.868
**BFM**	Males	16.6†	(10.8)	29.7**	(8.6)	0.002
(kg)	Females	21.9†	(10.6)	26.0†	(19.0)	0.084
**BFMI**	Males	5.2†	(3.5)	9.6**	(2.9)	0.002
(kg/m^2^)	Females	8.1†	(4.2)	10.0†	(7.0)	0.084

ULBC, University of Limerick Body Composition; CD, Crohn’s disease; BM, body mass; BMI, body mass index; LTM, lean tissue mass; LTMI, lean tissue mass index; ALTM, appendicular lean tissue mass; ALTMI, appendicular lean tissue mass; BFM, body fat mass; BFMI, body fat mass index. *p < 0.05 **p < 0.005 † not normally distributed (CD patients: Shapiro Wilk, *p* < 0.05; ULBC: Kolmogorov–Smirnov, *p* < 0.05). Data are presented as mean (SD) or median (IQR). CD subjects: n = 30, males n = 13, females n = 17. Age 20–73y. ULBC: n = 1606, males n = 683, females n = 923.

**Figure 1:**
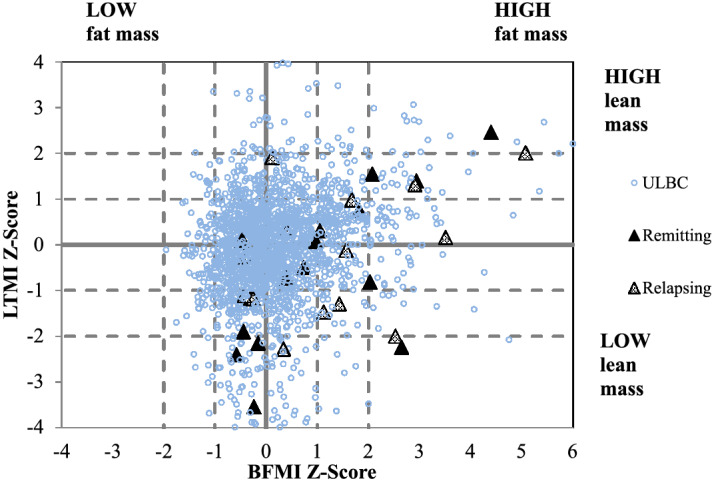
Age and sex matched Z-scores for BFMI versus LTMI for participants with remitting or relapsing CD and ULBC. LTMI, lean tissue mass index; BFMI, body fat mass index; ULBC, University of Limerick Body Composition Cohort. CD n = 30 (remitting n = 16, relapsing n = 14); age = 20-73y. ULBC (n = 1987); age = 18-81y.

When analysed individually (Fig. 1), it was evident that 13 (43%) of participants had aberrant body composition (LTMI Z-score ≤ − 2; BFMI Z-score ≥ 2). Of these, 12 (40%) had reduced LTMI (Z-score ≤ -1), of whom six exhibited very low LTMI (Z-score ≤ − 2). Two participants with very low LTMI (Z-score ≤ − 2) also exhibited very high BFMI (Z-score ≥ 2). Overall, nine participants (30%; 5 remitting, 4 relapsing) had very elevated fat mass defined by a BFMI Z-score ≥ 2. Notably, no participant had both low BFMI and high LTMI.

Throughout, the majority of BFM was located in the trunk or abdominal region; more specifically, trunk fat was primarily localised to the gynoid region as indicated by low mean android to gynoid ratio (0.6 (range: 0.24–1.35)). Two participants were excluded from bone density analysis due to osteoarthritis. Of the remaining 28 participants, one male (29y) had an L1–L4 Z-score below the expected range for age (− 2.7), and one female (72y) had a femoral neck T-score (− 2.7) indicative of osteoporosis.

### Dietary intake

**Figure 2:**
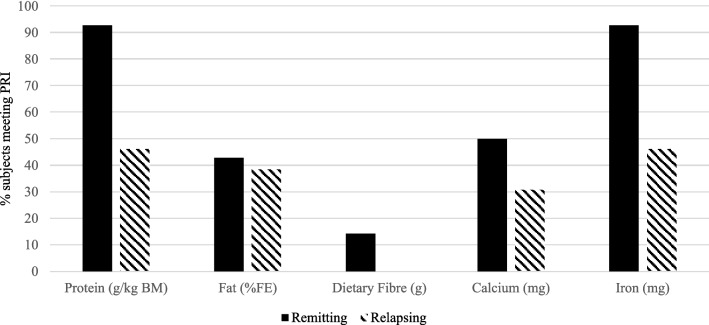
Proportion of participants in Crohn’s Disease remission (n = 14) or relapse (n = 13) who met the Population Reference Intake for protein, fat, dietary fibre, calcium and iron. BM, body mass; FE food energy; PRI, population reference intake; PRI: protein 0.83 g/kg/BM, total fat 20–35% FE, fibre 25 g/day, iron 11 (males) and 16 (females) mg/day (EFSA, 2015a, EFSA, 2015b, EFSA, 2015c EFSA, 2010a EFSA, 2010b).

Dietary records were completed by 27 participants. MDI of energy (kcal), protein (g), fat (g), carbohydrate (g) and iron (mg), reported in Table 3, were significantly lower in participants with relapsing CD (*p* < 0.05). Assessed according to Population Reference Intake (PRI), inadequate dietary protein intake was reported in a greater proportion of relapsing (54%) than remitting (7%) participants. All relapsing participants failed to achieve the PRI for fibre (Fig. 2). Similarly, more remitting than relapsing participants achieved the PRI for calcium (50% vs. 31%) and iron (93% *vs.* 46%).

**Table 3:** Comparison of the reported mean daily intake (energy and nutrients) of participants in Crohn’s Disease relapse or remission.

Nutrient	Remitting CD		Relapsing CD		*P value*
	*(n* = *14)*		*(n* = *13)*		
Energy (kcal)	2248*	(620)	1619*	(621)	0.014
Energy (kcal/kg BM)	28.5	(5.6)	22.7	(12.2)	0.121
Protein (g)	91.2*	(30.0)	60.3*	(29.1)	0.012
Protein (g/kg BM)	1.1	(0.2)	0.8	(0.5)	0.058
Fat (g)	91.6*	(31.2)	65.8*	(28.7)	0.035
Carbohydrate (g)	270*	(71)	200*	(72)	0.017
Dietary Fibre (g)	17.3	(7.2)	12.5	(6.2)	0.078
Protein (% FE)	16.4	(2.5)	14.8	(2.7)	0.128
Fat (%FE)	37.0	(5.6)	36.6	(5.5)	0.877
Carbohydrate (% FE)	46.3	(5.7)	48.3	(7.4)	0.441
Dietary Fibre (g/MJ TE)	1.9	(0.5)	1.8	(0.6)	0.906
Calcium (mg)	1094	(539)	875	(421)	0.252
Iron (mg)	16*†	(37)	8*†	(10)	0.011

CD, Crohn’s disease; BM, body mass; MUFA, monounsaturated fatty acids; PUFA, polyunsaturated fatty acids; FE, food energy; TE, total energy. * *p* < 0.05; † not normally distributed (Shapiro–Wilk *p* < 0.05). Remitting CD (n = 14, age 20–60y), relapsing CD (n = 13, age 29-73y). Data are presented as mean (SD) or median (IQR).

Physical activity monitoring was completed by 27 participants, 3 of whom were excluded for inadequate wear time. There was no difference between the TEE, physical activity level (PAL), mean daily step count, sedentary, light, moderate, vigorous and very vigorous physical activity levels between the two groups (Table 4). All participants exceeded population recommendations for physical activity (Department of Health, 2009; https://www.hse.ie/eng/about/who/healthwellbeing/our-priority-programmes/heal/heal-docs/the-national-guidelines-on-physical-activity-for-ireland.pdf).

**Table 4:** Estimated total energy expenditure (TEE), physical activity level (PAL), and mean daily activity rate and duration of participants in Crohn’s Disease relapse or remission.

	Remitting (n = 13)		Relapsing (n = 11)		*P value*
TEE (kcal)	3190	(838)	2870	(794)	0.350
PAL	1.8	(0.2)	1.7†	(0.3)	0.608
Step count (n)	8251	(3008)	6570	(3340)	0.208
Sedentary (h)	15.3	(2.3)	16.1	(2.4)	0.450
Light (h)	5.2	(1.3)	4.8	(1.3)	0.446
Moderate (h)	2.4	(1.1)	1.9†	(0.9)	0.569
Vigorous (h)	0.2†	(0.8)	0.1†	(0.8)	0.865
Very Vigorous (h)	0.0†	(0.0)	0.0†	(0.0)	0.955

PAL, physical activity level; h, hours. † not normally distributed (Shapiro–Wilk *p* < 0.05). Age 20-73y. Sedentary =  < 1.5 METs (Metabolic Equivalents); Light = 1.5–2.9 METs; Moderate = 3.0–5.9 METs; Vigorous = 6–8.9 METs; Very Vigorous =  ≥ 9 METs. Data are presented as mean (SD) or median (IQR).

## Discussion

Complementing recent data^27^, our findings support observations of reduced lean tissue mass (LTMI Z-score ≤ -2) and increased fat mass (BFMI Z-score ≥ 2) in 13 (43%) participants; very low LTMI (Z-score ≤ -2) was observed in 20% (n = 6) while nine participants (30%; 5 remitting, 4 relapsing) were identified as having very high fat mass (BFMI Z-score ≥ 2). Conversely, malnutrition with respect to protein, fat, fibre, calcium and iron was found in both remission and relapse, while physical activity exceeded population guidelines and did not appear to be affected by disease state. However, rare amongst studies published to date, we adopted an anthropometric approach in evaluating proportions of android and gynoid adiposity, observing that participants with CD, irrespective of relapse or remission, differed from a large representative healthy general population sample (ULBC) in exhibiting elevated gynoid fat and reduced android fat. CD is associated with mesenteric adipose tissue, or “creeping fat”, that envelops the affected intestine exclusive of other tissue; fat that is localised to the android region of the body. Our finding suggests, in the context of CD mesenteric adiposity specific to a region of overall reduced android fat, that CD may be a form of non-alcoholic fatty intestine disease. Of course, this suggestion is based on a relatively small study cohort. Definitive conclusions regarding this association between CD and fatty intestinal tissue will require additional study at a greater scale.

For many years, there has been a focus on understanding the relationship between BMI, obesity and Crohn’s disease^14,28,29^. In particular, emphasis has been placed on correlating childhood obesity with inflammatory bowel disease in attempting to discern patterns predictive of future illness^30^. More recently, these investigations have utilized improving imaging and body composition measurement technologies to concentrate specifically on the role of lean tissue and adipose tissue, especially visceral fat, in CD. These have included magnetic resonance imaging (MRI) computed tomography (CT) and, as used in our study, bioimpedance^31^ or DEXA^32^. As with our results, most studies determine levels of sarcopenia or relatively low lean tissue indices, combined with elevated adiposity in patients with CD, sometimes correlating with low skeletal mass^33^.

However, our study diverges from most previous reports in evaluating regional adiposity in participating patients. While there has been discussion of the impact of visceral fat in CD^18,32^, especially regarding potential impact on disease duration, severity, stricturing and risk of treatment complications^13^ and with more adverse outcomes^34^, we compared anthropometric measurements of participants with relapsing or remitting CD, finding no statistical difference between the groups, but observing considerable variation in regional adiposity between those with CD, irrespective of disease state, and a large body composition sample representative of the Irish general healthy population (similar, in fact, to that of the Austrian LEAD study)^35^. When looked at in the context of the suggested role of mesenteric adipose tissue in CD, this disparity between relatively reduced android adiposity and co-localised incidence of “creeping fat” is stark. More specifically, in light of preferential deposition of adipose tissue in hips and upper thighs of patients with CD, it is reasonable to argue that fat wrapping of inflamed intestinal tissue in the abdominal area deserves even greater attention than is currently focused. While mesenteric fat has been recognised as a potential barrier to inflammatory markers, to mitigate bacterial transposition and a reservoir of inflammatory adipokines^29,36^, we suggest tentatively that this juxtaposition of organ-specific and regional adiposity represents a rationale to refer to Crohn’s disease as a non-alcoholic fatty intestine condition, albeit that our data represent early insights limited to pilot-scale.

We further explored this observation; acknowledging the potential influence of nutrition on body composition we utilised a prospective dietary intake record, similar to approaches used previously^36^ that showed excessive dietary fat intake by all participants compared with the PRI, albeit that this appears to be reflective of a “Westernised” diet. Similarly, all relapsing participants failed to meet the PRI for fibre and, in the full cohort a high proportion (93%, n = 25) reported inadequate intake. Clinical advice typically prescribes a low fibre diet during episodes of relapsing and stricturing disease as many studies have shown that patients identify fibrous foods as exacerbating symptoms and increasing the risk of relapse.

In recognition of the importance of physical activity in preventing sarcopenia, obesity and general well-being in CD^37,38^ we employed wearable sensors to determine the physical activity levels of our study participants. Notably, while our initial expectation was that physical activity may be lower in the CD setting overall and especially in relapse^39^, our results showed a general level of activity in line with, or exceeding, that of the general population. We must, however, acknowledge a potential Hawthorne effect.

In summary, the reasons for aberrant body composition in CD are complex and multifaceted. In addition to lifestyle factors such as diet and physical activity, both the duration and active stage of the disease have been shown to correlate with elevated adiposity in particular. Treatments for the condition also play a role in altering body composition, as medications used for treatment of CD are known to result in fat mass gain. Furthermore, it remains important to classify body composition, as treatment prescription (specifically in relation to dietary interventions to optimise body mass and composition) as well as outcomes can be determined by aberrant body composition with implications for patient outcomes. In that light, we determined aberrant body composition, specifically low LTMI or high BFMI, in our outpatient cohort. More notably, anthropometric analysis showed that patients with CD differ from the general healthy population and exhibit elevated adiposity in their gynoid region despite the disease being associated with mesenteric fat exclusive to the android area of the body. As such then, it seems reasonable to propose further exploration of Crohn’s disease as a non-alcoholic fatty intestine condition, and to recommend investigation of diagnosic and therapeutic approaches in that context.

## Methods

### Participants

A convenience sample of 30 adult patients with CD, aged 20-73y (of whom 17 were female), was recruited from the Mid-West region of Ireland through the patient records of a specialist gastroenterology clinic at University of Limerick Hospital (UHL). Interested patients who were selected as eligible (adult, free-living and not pregnant or breast-feeding at the time of the study) volunteered to partake as study participants, and were placed on a waiting list for clinical examination.

Clinical examination included Crohn’s Disease Activity Index (CDAI)^24^, Harvey Bradshaw Index (HBI)^25^ and Montreal Classification^26^. Participants were classified as either in remission or relapse. Following clinical examination, participants attended three separate study visits. Visit 1 was scheduled within 1 month of the clinical examination to familiarise participants with the study format, study recording forms (e.g., weighed dietary record (WDR) detailed below), and measurement of physical activity (PA). Visit 2 was used to review the participants’ understanding of the study and their dietary and PA record-taking practices, and to discuss and resolve any recording problems encountered. The 7-day recording period commenced the day after Visit 2. At Visit 3, the a registered dietitian (RD) reviewed the returned records for accuracy and completeness. DEXA scans were performed on either Visit 1 or Visit 2.

This study was performed in line with the principles of the Declaration of Helsinki. Ethical approval was granted by the University Hospital Limerick Research Ethics Committee and the University of Limerick Education and Health Sciences Research Ethics Committee. Prior to commencement of the study, all participants were provided information regarding the study, associated risks and benefits. Each participant provided written, informed consent.

### Body composition

Height was measured to the nearest 0.1 cm using a stadiometer (Seca, Birmingham, UK) and body mass to the nearest 0.1 kg using a multi-frequency body composition analyzer based on bioelectrical impedance analysis (BIA) (Tanita MC-180MA, Tanita UK Ltd). BMI was calculated using the equation: mass (kg) divided by stature squared (m^2^).

Body composition was measured by DEXA (Lunar iDXA™, GE Healthcare, Chalfront St Giles and Bucks., UK) with enCORE™ v.14.1 and CoreScan software. DEXA scans were performed by either the primary author or other trained DEXA technicians. For standardisation, all segmental analyses were reviewed by the same DEXA technician. DEXA measures included total and segmental body fat mass (BFM, kg), % body fat (%BF), total and segmental lean tissue mass (LTM, kg), and total and segmental bone mineral content (BMC, kg). All procedures complied with the official positions of the International Society of Clinical Densitometry^40^.

Z-scores of lean tissue mass index (LTM/ht^2^) and body fat mass index (BFM/ht^2^) were created for each participant by subtracting their LTMI or BFMI from the median of their appropriate age and sex matched cohort of University of Limerick Body Composition Cohort (ULBC) and then dividing this difference by the standard deviation of the ULBC. The ULBC reference sample is representative of the Irish general healthy population and at the time of our study comprised the DEXA-determined body composition of volunteers aged 18–29y (males n = 616, females n = 412), 30–39y (males n = 102, females n = 99), 40–49y (males n = 47, females n = 76), 50–59y (males n = 70, females n = 258), 60–69y (males n = 59, females n = 214), 70–79y (males n = 4, females n = 30).

### Dietary analysis

A 7-day weighed dietary record (WDR) was used to collect food and beverage intake data. A weighing scale (DYMO®, Switzerland) was provided to each participant that was accurate to ± 1 g. These records were conducted prospectively with the support of a registered dietitian who provided verbal and written guidance. Food intake data were coded and analysed subsequently using WISP V4© (Tinuviel Software, Anglesey, UK).

Reported mean daily intakes (MDI) of energy, macro- and micronutrients were assessed for adequacy, for participants in relapse or remission, using European Food Safety Authority (EFSA) population reference intake (PRI) (EFSA; https://efsa.onlinelibrary.wiley.com/doi/toc/10.1002) and British Society of Gastroenterology recommendations for calcium intake in patients with CD (Lewis and Scott, 2007; https://gut.bmj.com/content/gutjnl/68/Suppl_3/s1.full.pdf). Furthermore, MDIs were compared between relapsing and remitting participants.

### Physical activity analysis

Total energy expenditure (TEE) was estimated using a portable SenseWear™ Mini Armband (BodyMedia Inc, Pittsburgh, PA). Each participants wore an armband for 7 days on the upper left arm (on the triceps at the mid-humerus point). The software (INNERVIEW version 8; Bodymedia, Pittsburgh, PA) provided percentages of on-body time. Days with on-body wear time < 90% (< 1296 min) were further examined; the day was included only if this off-body time was during 00:00–09:00 h as the software provides estimates for off-body time as 1 MET (metabolic equivalent), similar to what would be expected during sleep. If the 7-day SenseWear™ Mini record did not contain a minimum of 3 weekdays and 1 weekend day, the full record was excluded. Among the elements of PA included in the analysis were mean daily TEE, physical activity level (PAL) PAL = total energy expenditure (TEE) in a 24-h period, divided by basal metabolic rate (BMR), mean daily step count, time engaged in sedentary, light, moderate, vigorous and very vigorous physical activity levels (hours). Three participants were excluded from analysis—2 for inadequate SenseWear™ Mini wear time (1 male, 1 female), 1 female due to poor mobility associated with an unrelated medical condition.

### Statistical analysis

Statistical analysis was performed using IBM® SPSS® Version 22 (IBM Corporation, Armonk, NY). Data normality was assessed using a Shapiro–Wilk test. Data were reported as mean (standard deviation (SD)) or median (inter-quartile range (IQR)) if they were not normally distributed. Male and female participants were compared with the male and female cohorts of the ULBC using One-Sample t-tests; Wilcoxon signed rank tests were used if one or more group (i.e. CD or ULBC) was not normally distributed. The participants in this study, analysed as those in remission *vs.* relapse, were compared using Independent t-tests or Mann Whitney U tests, as appropriate, depending on the normality of the data distribution. A *p *value of < 0.05 (two-tailed) was considered to be statistically significant.
